# Supplementary material for: Distinct Neurodegenerative Pathways in Two NBIA Subtypes: Inflammatory Activation in C19orf12 but Not in PANK2 Mutation Carriers
Source: Cells. 2025 Nov 17;14(22):1801. doi: 10.3390/cells14221801 (PMC12651203; doi:10.3390/cells14221801)
Supplement: Supplementary file 1 [file cells-14-01801-s001.zip › cells-3961590-supplementary.pdf]

Spearman correlations in the control group												
	ICAM	MMP9	Esel	BDNF	Psel	S100B	α-syn	age	GFAP	NFL	Tau	UCH-L1
ICAM1		-0,171079	-0,107008	-0,112792	-0,201335	-0,105164	0,198687	-0,130329	0,079422	-0,162661	0,091445	0,045606
MMP9	-0,171079		0,391324	-0,074527	0,149277	0,085089	-0,057019	0,033252	-0,143938	-0,061638	-0,113250	0,232481
Es	-0,107008	0,391324		0,532814	0,643159	0,696456	0,227071	0,516628	0,286318	0,250111	0,193792	0,452280
BDNF	-0,112792	-0,074527	0,532814		0,443382	0,809605	0,214010	0,759209	0,224472	0,705385	0,267438	0,373081
Ps	-0,201335	0,149277	0,643159	0,443382		0,519434	0,009545	0,332517	0,243604	-0,015799	0,027367	0,332592
S100B	-0,105164	0,085089	0,696456	0,809605	0,519434		0,217902	0,777813	0,183638	0,485780	0,131413	0,478372
α-syn	0,198687	-0,057019	0,227071	0,214010	0,009545	0,217902		0,236853	-0,029640	0,270712	0,099480	0,222048
AGE	-0,130329	0,033252	0,516628	0,759209	0,332517	0,777813	0,236853		0,127428	0,415738	0,050441	0,390986
GFAP	0,079422	-0,143938	0,286318	0,224472	0,243604	0,183638	-0,029640	0,127428		0,253227	0,473468	0,265406
NfL	-0,162661	-0,061638	0,250111	0,705385	-0,015799	0,485780	0,270712	0,415738	0,253227		0,479804	0,420338
Tau	0,091445	-0,113250	0,193792	0,267438	0,027367	0,131413	0,099480	0,050441	0,473468	0,479804		0,384470
UCH-L1	0,045606	0,232481	0,452280	0,373081	0,332592	0,478372	0,222048	0,390986	0,265406	0,420338	0,384470	

Spearman correlation in the MPAN group																		
	ICAM	MMP9	Es	BDNF	Ps	S100B	α-syn	AGE	GFAP	NFL	Tau	UHC-L1	DRS1	DRS2	DRS3	DRS4	DRS5	DRS6
ICAM1		-0,065385	0,576923	-0,050769	-0,010000	0,080769	-0,116154	0,048687	0,129231	0,224615	0,210040	0,159231	0,198653	0,275345	-0,126036	0,127726	0,265456	0,074403
MMP9	-0,065385		0,090000	0,184615	0,074615	0,143077	0,237692	0,122876	-0,340769	0,063077	0,228505	0,293846	0,204812	0,191544	0,178455	0,270887	-0,055989	0,107556
Es	0,576923	0,090000		-0,015385	0,402308	-0,107692	-0,195385	0,362060	-0,028462	0,102308	0,378150	0,253077	0,391146	0,379227	0,029293	0,237316	0,243531	0,222051
BDNF	-0,050769	0,184615	-0,015385		0,304615	-0,011538	0,077692	0,081531	-0,057692	-0,170769	-0,256588	0,236923	-0,199038	-0,055223	-0,228561	-0,110747	0,068909	-0,274480
Ps	-0,010000	0,074615	0,402308	0,304615		-0,261538	-0,159231	0,087713	-0,371538	0,144615	-0,303520	-0,066923	0,177863	-0,040935	-0,110619	0,112291	0,310482	0,106400
S100B	0,080769	0,143077	-0,107692	-0,011538	-0,261538		0,906923	-0,182382	-0,350769	-0,367692	0,170033	0,193846	-0,268720	-0,126666	-0,060513	0,102258	-0,392703	-0,112182
α-syn	-0,116154	0,237692	-0,195385	0,077692	-0,159231	0,906923		-0,045982	-0,310000	-0,225385	0,146182	0,243077	-0,117421	0,007337	-0,012719	0,227283	-0,281900	0,034310
AGE	0,048687	0,122876	0,362060	0,081531	0,087713	-0,182382	-0,045982		0,103556	0,265845	0,320969	0,383312	0,682271	0,783123	0,458085	0,641792	0,631127	0,729088
GFAP	0,129231	-0,340769	-0,028462	-0,057692	-0,371538	-0,350769	-0,310000	0,103556		0,210000	0,153491	0,342308	0,202118	0,246768	0,161881	0,080263	0,142516	0,155359
NfL	0,224615	0,063077	0,102308	-0,170769	0,144615	-0,367692	-0,225385	0,265845	0,210000		-0,089633	-0,116154	0,567084	0,361463	0,071305	0,204130	0,647979	0,283732
Tau	0,210040	0,228505	0,378150	-0,256588	-0,303520	0,170033	0,146182	0,320969	0,153491	-0,089633		0,177726	0,196766	0,409234	0,203740	0,269781	0,072055	0,133411
UCH-L1	0,159231	0,293846	0,253077	0,236923	-0,066923	0,193846	0,243077	0,383312	0,342308	-0,116154	0,177726		0,199808	0,393902	0,344961	0,509360	0,039544	0,450271
DRS1	0,198653	0,204812	0,391146	-0,199038	0,177863	-0,268720	-0,117421	0,682271	0,202118	0,567084	0,196766	0,199808		0,790495	0,561343	0,684050	0,660556	0,767896
DRS2	0,275345	0,191544	0,379227	-0,055223	-0,040935	-0,126666	0,007337	0,783123	0,246768	0,361463	0,409234	0,393902	0,790495		0,562501	0,779930	0,630956	0,756727
DRS3	-0,126036	0,178455	0,029293	-0,228561	-0,110619	-0,060513	-0,012719	0,458085	0,161881	0,071305	0,203740	0,344961	0,561343	0,562501		0,644432	0,413939	0,682828
DRS4	0,127726	0,270887	0,237316	-0,110747	0,112291	0,102258	0,227283	0,641792	0,080263	0,204130	0,269781	0,509360	0,684050	0,779930	0,644432		0,520479	0,784568
DRS5	0,265456	-0,055989	0,243531	0,068909	0,310482	-0,392703	-0,281900	0,631127	0,142516	0,647979	0,072055	0,039544	0,660556	0,630956	0,413939	0,520479		0,563340
DRS6	0,074403	0,107556	0,222051	-0,274480	0,106400	-0,112182	0,034310	0,729088	0,155359	0,283732	0,133411	0,450271	0,767896	0,756727	0,682828	0,784568	0,563340	

Spearman correlation in the PKAN group																		
	ICAM	MMP9	Es	BDNF	Ps	S100B	α-syn	AGE	GFAP	NFL	Tau	UHC-L1	DRS1	DRS2	DRS3	DRS4	DRS5	DRS6
ICAM1		0,090909	0,545455	-0,111888	0,048951	-0,279720	-0,342657	-0,183429	-0,097902	-0,293706	0,279720	0,314685	0,746474	-0,117803	0,317930	-0,249564	0,363330	0,017606
MMP9	0,090909		-0,069930	0,846154	0,755245	-0,671329	0,146853	0,610253	-0,069930	-0,083916	-0,048951	0,132867	-0,090482	0,154617	-0,182185	0,442888	-0,059967	-0,126764
Es	0,545455	-0,069930		-0,335664	0,006993	-0,244755	-0,209790	-0,031747	-0,237762	0,006993	-0,111888	-0,076923	0,237514	-0,007363	0,092878	-0,555368	-0,218703	-0,281697
BDNF	-0,111888	0,846154	-0,335664		0,650350	-0,587413	0,300699	0,645528	0,013986	-0,027972	-0,076923	0,356643	-0,241284	0,279783	-0,171468	0,541308	-0,014110	-0,172539
Ps	0,048951	0,755245	0,006993	0,650350		-0,482517	0,223776	0,652583	0,307692	0,377622	0,195804	0,496503	0,045241	-0,382860	0,189329	0,502643	0,250451	0,154933
S100B	-0,279720	-0,671329	-0,244755	-0,587413	-0,482517		0,216783	-0,236341	-0,118881	0,083916	-0,167832	-0,167832	-0,011310	-0,257695	-0,082162	0,052725	-0,144626	0,024648
α-syn	-0,342657	0,146853	-0,209790	0,300699	0,223776	0,216783		0,278670	0,293706	0,587413	0,153846	0,230769	-0,316686	-0,066264	-0,521548	0,108965	-0,116407	-0,169018
AGE	-0,183429	0,610253	-0,031747	0,645528	0,652583	-0,236341	0,278670		-0,275143	0,215176	-0,437407	0,239868	-0,346115	0,051995	-0,277500	0,528372	-0,306050	-0,444050
GFAP	-0,097902	-0,069930	-0,237762	0,013986	0,307692	-0,118881	0,293706	-0,275143		0,685315	0,832168	0,391608	0,203584	-0,633192	0,242913	0,123025	0,740770	0,672552
NfL	-0,293706	-0,083916	0,006993	-0,027972	0,377622	0,083916	0,587413	0,215176	0,685315		0,349650	0,244755	-0,128182	-0,574291	-0,189329	0,137085	0,211648	0,102115
Tau	0,279720	-0,048951	-0,111888	-0,076923	0,195804	-0,167832	0,153846	-0,437407	0,832168	0,349650		0,461538	0,512730	-0,574291	0,389375	-0,137085	0,878341	0,785231
UCH-L1	0,314685	0,132867	-0,076923	0,356643	0,496503	-0,167832	0,230769	0,239868	0,391608	0,244755	0,461538		0,226204	-0,301871	0,521548	0,151144	0,617308	0,264091
DRS1	0,746474	-0,090482	0,237514	-0,241284	0,045241	-0,011310	-0,316686	-0,346115	0,203584	-0,128182	0,512730	0,226204		-0,539836	0,446802	-0,030320	0,600947	0,451809
DRS2	-0,117803	0,154617	-0,007363	0,279783	-0,382860	-0,257695	-0,066264	0,051995	-0,633192	-0,574291	-0,574291	-0,301871	-0,539836		-0,383632	-0,299766	-0,638801	-0,652497
DRS3	0,317930	-0,182185	0,092878	-0,171468	0,189329	-0,082162	-0,521548	-0,277500	0,242913	-0,189329	0,389375	0,521548	0,446802	-0,383632		-0,087983	0,605454	0,631360
DRS4	-0,249564	0,442888	-0,555368	0,541308	0,502643	0,052725	0,108965	0,528372	0,123025	0,137085	-0,137085	0,151144	-0,030320	-0,299766	-0,087983		0,138299	0,092036
DRS5	0,363330	-0,059967	-0,218703	-0,014110	0,250451	-0,144626	-0,116407	-0,306050	0,740770	0,211648	0,878341	0,617308	0,600947	-0,638801	0,605454	0,138299		0,811724
DRS6	0,017606	-0,126764	-0,281697	-0,172539	0,154933	0,024648	-0,169018	-0,444050	0,672552	0,102115	0,785231	0,264091	0,451809	-0,652497	0,631360	0,092036	0,811724	
